# Study of Pavement Micro- and Macro-Texture Evolution Due to Traffic Polishing Using 3D Areal Parameters

**DOI:** 10.3390/ma14195769

**Published:** 2021-10-02

**Authors:** Yiwen Zou, Guangwei Yang, Wanqing Huang, Yang Lu, Yanjun Qiu, Kelvin C. P. Wang

**Affiliations:** 1 School of Civil Engineering, Southwest Jiaotong University, Chengdu 610031, China; luyang@home.swjtu.edu.cn (Y.L.); publicqiu@vip.163.com (Y.Q.); 2 School of Civil and Environmental Engineering, Oklahoma State University, Stillwater, OK 74078, USA; wangchenping@swjtu.cn; 3 Sichuan Communication Surveying & Design Institute Co., Ltd., Chengdu 610041, China; hangwq1978@163.com

**Keywords:** traffic polishing, pavement micro-texture, pavement macro-texture, high-resolution 3D texture image, 3D areal texture parameters

## Abstract

Pavement micro- and macro-texture have significant effects on roadway friction and driving safety. The influence of traffic polish on pavement texture has been investigated in many laboratory studies. This paper conducts field evaluation of pavement micro- and macro-texture under actual traffic polishing using three-dimensional (3D) areal parameters. A portable high-resolution 3D laser scanner measured pavement texture from a field site in 2018, 2019, and 2020. Then, the 3D texture data was decomposed to micro- and macro-texture using Fourier transform and Butterworth filter methods. Twenty 3D areal parameters from five categories, including height, spatial, hybrid, function, and feature parameters, were calculated to characterize pavement micro- and macro-texture. The results demonstrate that the 3D areal parameters provide an alternative to comprehensively characterize the evolution of pavement texture under traffic polish from different aspects.

## 1. Introduction

### 1.1. Background

Pavement skid resistance has positive effects on reducing traffic accidents in dry or wet conditions [1]. For example, the skidding risk would increase rapidly when the pavement skid number (SN) from locked-wheel skid testers is below 50 and decrease significantly when the value of SN is over 65 [2]. Additionally, traffic accidents increased 60% when the value of SN decreased from 48 to 33 [3]. Therefore, it is critical to design pavements surface with high skid resistance and wear resistance to ensure driving safety during design life [4].

Skid resistance of pavement surface varies under traffic polishing throughout the design life: It typically increases to a peak at the initial stage and decreases continuously at the following stages of pavement life [5,6]. However, the level of skid resistance is dependent on the wear process of pavement surface texture [7], as the skid resistance of pavement comes directly from the contact between vehicle tires and pavement surface micro- and macro- texture [8]. Pavement surface texture is recognized as the dominating factor influencing pavement skid resistance [9].

Therefore, many studies have been performed to investigate how traffic wear affects pavement texture over time in order to evaluate pavement skid resistance under traffic polish. Pavement texture can be categorized into macro-texture (0.5 mm < wavelength < 50 mm) and micro-texture (wavelength < 0.5 mm) based on the wavelength of its components [10]. The pavement macro-texture provides drainage channels when it rains and comprises the hysteretic component of friction, while the pavement micro-texture provides actual contact with the tire and comprises the adhesion part of friction [11].

Typically, measurement of pavement macro-texture adopts the sand patch method, the outflow meter, or the circular texture meter (CTM) using a two-dimensional (2D) texture profile [12]. Indicators like mean texture depth (MTD), mean profile depth (MPD), or root mean square depth (RMSD) is customarily applied to characterize pavement macro-texture [13]. Besides, the pavement micro-texture is evaluated by indirect friction measurement devices testing at low speed, such as the British portable tester (BPT), the dynamic friction tester (DFT), and the locked-wheel skid trailer [12].

Recently, pavement surface micro-texture was measured with high-resolution cameras in the laboratory to obtain more texture details [14]. Moreover, the advanced high-resolution laser device can conveniently collect three-dimensional (3D) surface texture data from the field and achieve enough accuracy to characterize pavement micro- and macro-texture [15]. The acquisition of high-resolution surface texture information can significantly assist the investigation of the micro- and macro-texture contributions to skid resistance [16].

Further, 3D areal surface texture parameters have been utilized extensively in modern manufacturing industries to control and evaluate the surface finishing of products [17]. The 3D areal texture parameters contain aspects of surface height, spatial, hybrid, function, and feature information, whereas the traditional parameters only contain height information [17]. The areal texture parameters can characterize surface texture functionality and understand texture characteristics in different perspectives that the traditional texture parameter fails to achieve [18]. Therefore, some recent studies attempted to evaluate pavement texture using 3D areal parameters and correlate them with skid resistance [19,20,21]. The study described in this paper used 3D areal parameters to evaluate pavement texture changes under traffic loading.

Many studies have been performed to investigate how traffic polish affects pavement texture over time so that pavement can be constructed with desired texture features to maintain good skid resistance [22]. Several devices were developed to study the wear-resisting feature of pavements in the laboratory, such as the Wehner/Schulze device (W/S) [23], the Aachen polishing machine (APM) [24], and other accelerated polishing machines [25,26,27,28]. These devices evaluate the evolution of pavement texture under simulated traffic polishing in controlled laboratory conditions rather than actual traffic polishing from various vehicles.

Further, Kane et al. proposed a polishing model to predict the surface variation with polishing cycles based on laboratory testing using the W/S machine and adopted the roughness parameters (R_q_) to validate the model [29]. Druta et al. conducted accelerated polish testing on stone matrix asphalt (SMA) species and found that MPD had completely different changing trends with BPN during the polish process [30]. Wang et al. tried to quantify the effect of aggregate size with W/S device on polishing resistance, and the texture variation characterized by power spectral density (PSD) showed that the coarser aggregate had a significantly rougher texture [5]. Wu and Abadie simulated the wearing process with an accelerated polishing machine and measured MPD with a CTM, and the results indicated that the MPD values tended to remain constant under different polishing cycles [31]. Plati and Pomoni investigated the long-term field data of skid resistance and macro-texture and found that the MPD and grip number (GN) presented a contrary trend under traffic polish [32].

### 1.2. Research Need

Several limitations in previous studies have been identified and summarized as follows:

(1) Many previous studies evaluated pavement wear performance in the laboratory using polishing machines. In laboratory studies, it is challenging to repeat the actual pavement polish process in the field involving traffic polishing from various vehicle types under different environmental conditions such as temperature, precipitation, or freeze-thaw cycles.
(2) In previous studies, 2D texture profiles were typically collected at the macro-texture level to evaluate pavement texture variation under traffic polish. With the advancement of pavement data collection, high-resolution 3D texture data should be applied to understand better the evolution of asphalt pavement micro- and macro-texture under traffic polish.
(3) Traditional pavement texture parameters only consider texture height distribution while miss other texture characteristics (such as spatial, hybrid, and so on). Hence, different categories of 3D areal parameters should be explored to characterize pavement micro- and macro-texture under traffic polishing from different aspects.

Therefore, it is necessary to conduct field studies to understand pavement micro- and macro-texture evolution under actual traffic polish using different 3D areal texture parameters.

### 1.3. Objective

In this study, a field asphalt pavement site was monitored from 2018 to 2020 to study the influence of traffic polish on the evolution of pavement micro- and macro-texture. Pavement 3D texture images were collected using an LS-40 3D laser scanner (HyMIT Measurement Instrument Technology, Austin, TX, USA) for three years. Then, pavement micro- and macro-texture were separated from the obtained 3D texture data using two-dimensional discrete Fourier Transform method and Butterworth filters method 21. Next, twenty 3D areal parameters from five categories (height, spatial, hybrid, function, and feature) were calculated for pavement 3D micro- and macro-texture. The obtained micro- and macro-texture parameters in three years were analyzed to describe the evolution of surface texture under actual traffic polish from different perspectives.

## 2. Field Data Collection

This study selected the field site on a suburb road paved with dense-graded asphalt mixture (HMA-13) in Yongning avenue, located in an industrial district of Chengdu, China. There were about 3 million passage cars on this site in 2019, and the traffic volume had an 11.9% growth in 2020. The site was constructed in 2018 and monitored until 2020 for pavement texture variations under actual traffic polishing.

**Figure 1:**
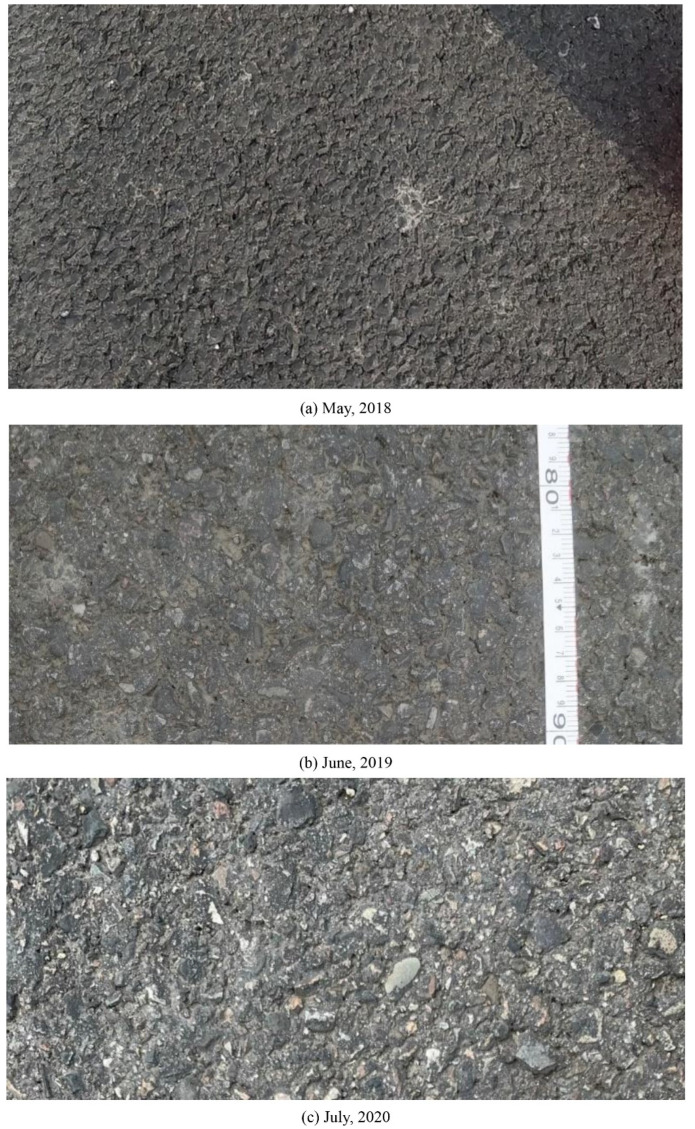
Pavement surface pictures from the field site: (**a**) May 2018, (**b**) June 2019, and (**c**) July 2020.

Figure 1 shows example pavement images from this site in 2018, 2019, and 2020, individually. Most of the aggregates were coated with bitumen in 2018 when the pavement was just constructed (Figure 1a). After traffic polish from 2018 to 2019, the bitumen layer was gradually removed, and the coarse aggregate was exposed to field environmental effect and traffic polishing and compacting, as shown in Figure 1b. Further, the texture of coarse aggregates in 2020 looked smoother and more aging than that in 2019 due to traffic polish (see Figure 1c).

**Figure 2:**
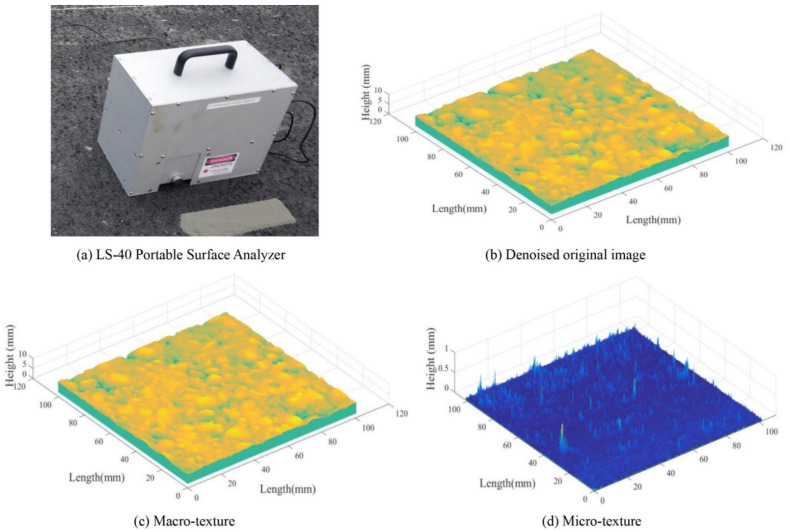
LS-40 and examples 3D texture data: (**a**) LS-40 Portable Surface Analyzer, (**b**) Denoised original image, (**c**) Macro-texture, and (**d**) Micro-texture.

Figure 2a shows the LS-40 Portable Surface Analyzer (LS-40, HyMIT Measurement Instrument Technology, Austin, TX, USA) that was used to record 3D texture images on this site to quantify pavement texture evolution due to traffic polish. The LS-40 scans a 102.4 × 102.4 mm pavement surface area with height resolution (z) at 0.01 mm and lateral resolution (x, y) at 0.05 mm. From 2018 to 2020, a total number of 42 3D texture images were obtained from the wheel path during each data collection from previously marked locations on this site.

The 3D texture data collected by LS-40 was denoised (see Figure 2b) by a Gaussian smoothing filter with a kernel size of 5 × 5. Then the Fourier transform converted the texture height data into the frequency domain, and the Butterworth filter separated texture components in the frequency domain into micro-texture and macro-texture at a boundary of 2 Hz. Subsequently, the inverse Fourier transform converted the frequency domain micro-texture and macro-texture data back to texture height data, respectively, as shown in Figure 2c,d. The detailed procedure of texture data processing was published in a previous research [21].

**Figure 3:**
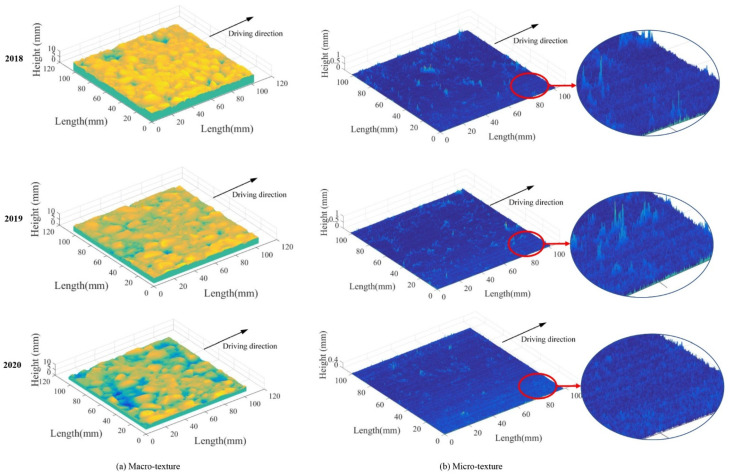
Evolution of pavement micro- and macro-texture: (**a**) Macro-texture, and (**b**) Micro-texture.

Figure 3 shows an example of how 3D pavement micro- and macro-texture changed from 2018 to 2020. It is noteworthy that the height of macro-texture was decreasing over time and tiny stripes formed on the micro-texture along driving direction in 2020. To characterize the evolution of micro- and macro-texture, quantitative analysis is conducted in the following section via 3D areal parameters.

## 3. Three Dimensional Areal Parameters

In this section, twenty 3D areal texture parameters from five categories (height, spatial, hybrid, functional, and feature) were calculated for both 3D micro- and macro-texture to investigate the evolution of pavement surface texture under actual traffic polishing and environmental impacts. The category, name, and unit of these 3D areal parameters are summarized in Table 1. The detailed definition of these parameters is introduced as follows.

### 3.1. Height Parameters

The height parameters consider the surface height information, but neglect the horizontal input. In this section, four height parameters, including arithmetic mean height (S_a_), root mean square height (S_q_), skewness (S_sk_), and kurtosis (S_ku_), were calculated per Equations (1)–(4) [10]. The S_a_ and S_q_ measure the overall height of a surface and correlate intensely with each other, and the S_sk_ and S_ku_ describe the shape of the surface probability density [10]. The S_sk_ indicates the symmetry of the height probability density curve, and the S_ku_ characterizes the kurtosis of the probability density curve. Pavement surface with positive S_sk_ would have spike structure, and surface with negative S_sk_ would have valley structure. Moreover, a higher S_ku_ implies more significant height variation of surface peaks or valleys. Significantly, the S_sk_ is 0.0 and the S_ku_ is 3.0 when the surface probability density function is Gaussian distribution [33].

(1) $$S_{a}=\sqrt{\frac{1}{A}\iint \left|z\left(x,y\right)\right|\mathrm{dxdy}}$$

(2) $$S_{q}=\sqrt{\frac{1}{A}\iint z{\left(x,y\right)}^{2}\;\mathrm{dxdy}}$$

(3) $$S_{\mathrm{sk}}=\frac{1}{{A\;S}_{q}^{3}}\iint z{\left(x,y\right)}^{3}\;\mathrm{dxdy}$$

(4) $$S_{\mathrm{ku}}=\frac{1}{{\mathrm{AS}}_{q}^{4}}\iint z{\left(x,y\right)}^{4}\;\mathrm{dxdy}$$

where A is the area of a 3D image; z is the height value of pixels in a 3D image; x and y are the horizontal coordinates of pixels in a 3D image.

### 3.2. Spatial Parameters

**Figure 4:**
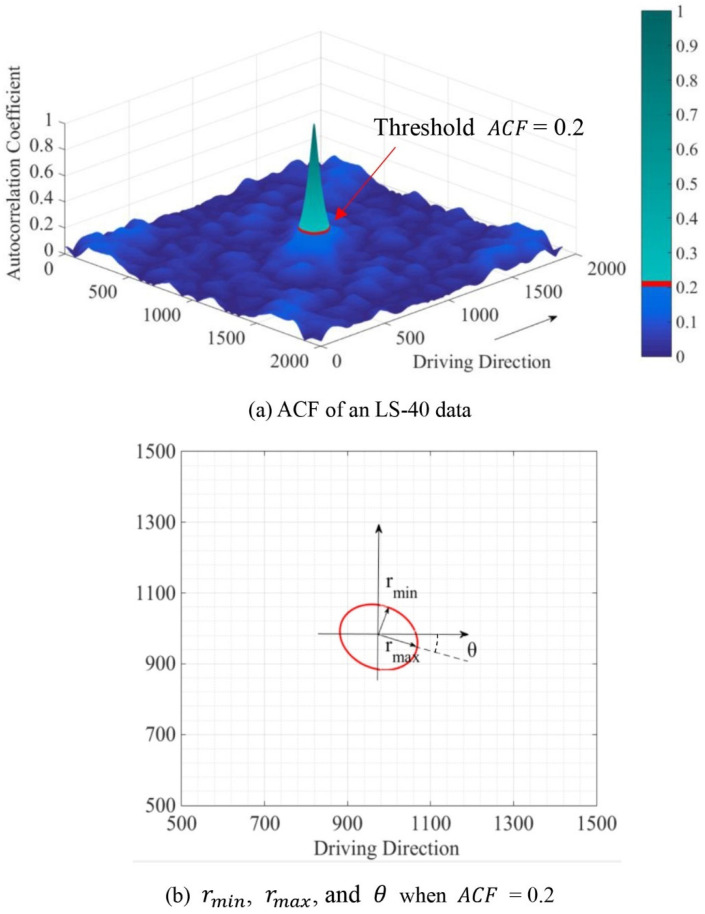
Calculation of spatial parameters: (**a**) ACF of an LS-40 data, and (**b**) r_min_, r_max_, and θ when ACF = 0.2.

The calculation of spatial parameters involves the autocorrelation function (ACF) of a 3D texture surface. The ACF calculates the similar degree of a surface z(x, y) and the duplicate surface z(x-τ_x_, y-τ_y_) with a horizontal shift (τ_x_, τ_y_) [17]. Equation (2) shows the function to calculate ACF, and Figure 4a shows the ACF of an obtained LS-40 data as an example. For instance, the ACF is 1.0 when the LS-40 data has a horizontal shift (0, 0), the ACF equals 0.2 when the LS-40 data has a horizontal change along the red circle highlighted in Figure 4a.

The spatial parameters, including autocorrelation length (S_al_), texture aspect ratio (S_tr_), and texture direction (S_td_), were obtained for each LS-40 data per Equations (5)–(8) using 0.2 as the threshold of ACF. Figure 4b illustrates an example of how to calculate $r_{\min}$, $r_{\max}$, and θ from the red circle when ACF = 0.2 [10]. The S_al_ is defined as the horizontal distance $r_{\min}$ that has the fastest decay to ACF = 0.2 [34]. Additionally, the S_tr_ is calculated as the ratio of the fastest decay distance r_min_ to the slowest decay distance r_max_, which is the most critical indicator to characterize isotropy of surface texture in the horizontal direction. The surface is isotropic when S_tr_ equals 1, and the surface is anisotropic when S_tr_ equals 0. Further, the S_td_ gives the angle of r_max_ for a surface texture, as shown in Figure 4b.

(5) $$\mathrm{ACF}\left(\tau_{x},\tau_{y}\right)=\frac{\iint z\left(x,y\right)z\left(x-\tau_{x},y-\tau_{y}\right)\mathrm{dxdy}}{\iint z^{2}\left(x,y\right)\mathrm{dxdy}}$$

(6) $$S_{\mathrm{al}}=\underset{\tau_{x},{\;\tau }_{y}\in R\;}{\min}\sqrt{\tau_{x}^{2}+\tau_{y}^{2}}=r_{\min}$$

where $R=\left\{\left(\tau_{x},\tau_{y}\right):\mathrm{ACF}\left(\tau_{x},\tau_{y}\right)\leq 0.2\right\}$.

(7) $$S_{\mathrm{tr}}=\frac{r_{\min}}{r_{\max}}\;$$

(8) $$S_{\mathrm{td}}=\theta$$

where τ_x_ and τ_y_ are the shifting along and perpendicular to the driving direction, respectively; r_max_ and r_min_ are the slowest and fastest decay distance to ACF = 0.2, respectively; θ is the angle between r_max_ and driving direction.

### 3.3. Hybrid Parameters

The hybrid parameters describe the height and spacing information of a surface texture. They measure the angular slope of the 3D profile and are handy for assessing friction, adhesion, vibration, etc. The root mean square gradient (S_dq_) and developed interfacial area ratio (S_dr_) were calculated per Equations (9) and (10) as the hybrid parameters based on the surface local gradient. The S_dq_ and S_dr_ can be utilized to assess surface cosmetic flatness and correlate to adhesion property [35]. A flat surface would have both S_dq_, and S_dr_ value equals 0. Besides, a 45° inclined surface would have a S_dq_ value of 1 and a S_dq_ value of 41.4%.

(9) $$S_{\mathrm{dq}}=\sqrt{\frac{1}{A}\iint {\left(\frac{\partial z}{\partial x}\right)}^{2}+{\left(\frac{\partial z}{\partial y}\right)}^{2}\mathrm{dxdy}}$$

(10) $$S_{\mathrm{dr}}=\frac{1}{A}\{\iint \left[\sqrt{1+{\left(\frac{\partial z}{\partial x}\right)}^{2}+{\left(\frac{\partial z}{\partial y}\right)}^{2}}-1\right]\mathrm{dxdy}\}\times 100\%$$

### 3.4. Function Parameters

**Figure 5:**
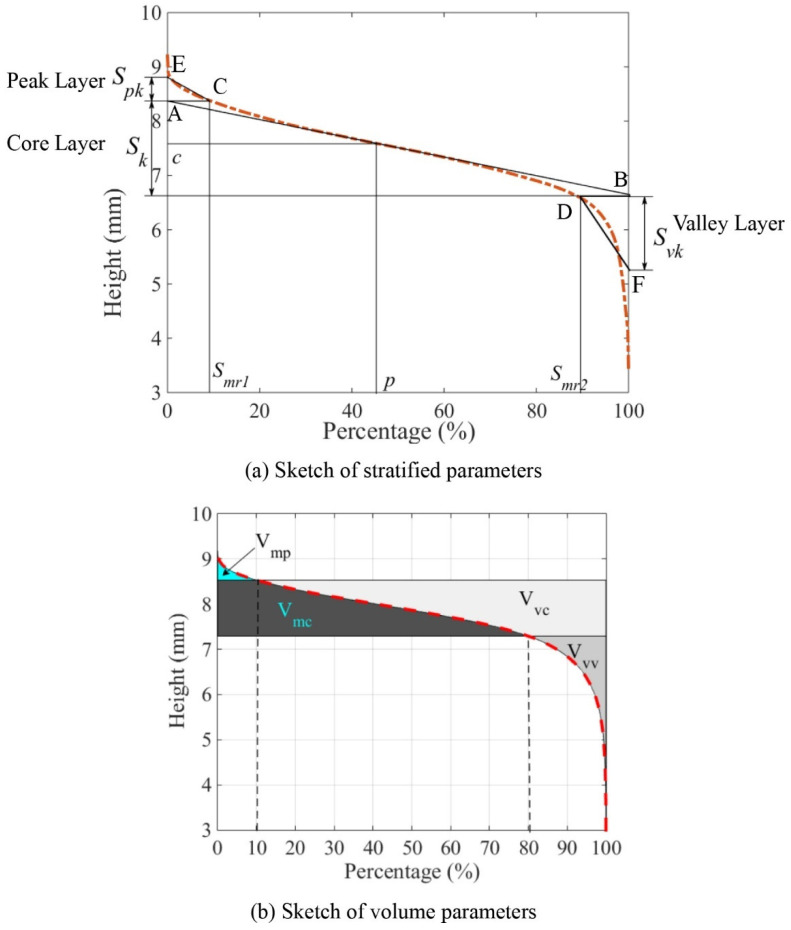
Calculation of function parameters: (**a**) Sketch of stratified parameters, and (**b**) Sketch of volume parameters.

The function parameters are strongly associated with surface functions, such as wearing, bearing, and hydroplaning. Three sub-categories of functional parameters, material ratio, stratified, and volume, were calculated for 3D micro- and macro-texture data based on the cumulative height distribution curve or material ratio curve. The dashed line in Figure 5 shows examples of the cumulative height curve of the 3D texture. The ordinate is the surface height, and the abscissa is the cumulative probability above a certain height. The function parameters characterize peak, core, and valley features of pavement micro- and macro-texture. Details of how to calculate stratified and volume parameters per the cumulative height curve are introduced as follows.

#### 3.4.1. Material Ratio Parameters

The areal material ratio parameters employ the peak extreme height (S_xp_) and the surface section difference (S_dc_) to characterize the upper half part and the general height of a surface, respectively [18]. The calculation of S_xp_ considers the surface part among the mean plane (50%) and the summit (2.5%). The parameter S_dc_ defines the general height difference of the surface without taking the highest peaks (below 2%) and the lowest valleys (above 98%) into account, as shown in Equations (11) and (12).

(11) $$S_{\mathrm{xp}}=S_{\mathrm{mc}}\left(2.5\%\right)-S_{\mathrm{mc}}\left(50\%\right)$$

(12) $$S_{\mathrm{dc}}=S_{\mathrm{mc}}\left(2\%\right)-S_{\mathrm{mc}}\left(98\%\right)$$

where $S_{\mathrm{mc}}\left(p\right)$ is the height value c corresponding to a material ratio p in Figure 5a.

#### 3.4.2. Stratified Parameters

According to the cumulative height distribution curve, the surface topography is stratified into three parts: peak layer, core layer, and valley layer. The three parts of a surface texture are represented by reduced peak height (S_pk_), core height (S_k_), and reduced dale height (S_vk_), as shown in Figure 5a [10]. The calculation of stratified parameters in this study requires the following steps, as illustrated in Figure 5a:

- First, a straight line is plotted tangent to the middle part of the dashed cumulative height distribution curve. The tangent line intersects the vertical axes of percentage 0% and 100% at two points, A and B. The corresponding height of A and B are peak and valley thresholds.
- Then, points C and D are projected on the cumulative height distribution curve for heights A and B to define percentages of Smr1 and Smr2. The height difference of A and B is defined as S_k_.
- The area enclosed below the cumulative height distribution curve and above AC is represented by the triangle ACE that has an equivalent area. The height difference of E and A is defined as S_pk_.
- The area enclosed above the cumulative height distribution curve and below BD is represented by the triangle BDF that has an equivalent area. The height difference of F and B is defined as S_vk_.

Generally, the S_pk_ measures the equivalent height of the surface summit, which is the primary and the most worn surface height. The S_k_ evaluates the long-term contact height of a surface. The S_vk_ measures the equivalent height of deep grooves, which would hold debris from the upper surface [36].

#### 3.4.3. Volume Parameters

The peak material volume (V_mp_), core material volume (V_mc_), core void volume (V_vc_), and dales void volume (V_vv_) were calculated as per Equations (13)–(16) [10] as volume parameters and illustrated in Figure 5b. The material ratios, 10% and 80%, are specified as thresholds of the accumulated height to define peak and void of a surface texture [10]. The V_mp_ represents the material volume that is most likely to be removed by traffic polish. Moreover, the V_mc_ measures the material volume polished by traffic but not as much as the V_mp_ is. The V_vc_ is the surface void volume opposite to the V_mc_. The V_vv_ indicates the void volume with a cumulative height distribution of the lowest 20%.

(13) $$V_{\mathrm{mp}}=V_{m}\left(10\%\right)$$

(14) $$V_{\mathrm{mc}}=V_{m}\left(80\%\right)-V_{m}\left(10\%\right)$$

(15) $$V_{\mathrm{vc}}=V_{v}\left(10\%\right)-V_{v}\left(80\%\right)$$

(16) $$V_{\mathrm{vv}}=V_{v}\left(80\%\right)$$

where V_m_(mr) is material volume above the height corresponding to a material ratio mr to the highest peak; V_v_(mr) is void volume below the height corresponding to a material ratio mr to the lowest valley.

As shown in Figure 5, stratified parameters and volume parameters divide the surface texture into peak, core, and valley with a different method based on the cumulative height distribution curve. To define surface peak and valley, volume parameters use 10% and 80% material ratios, whereas stratified parameters utilize the tangent line of the cumulative height distribution curve to determine mr1 and mr2. Further, volume parameters calculate these layers’ material or void volume, and the stratified parameters estimate equivalent height for surface peak or valley layer.

### 3.5. Feature Parameters

The feature parameters can be used to characterize specified features of surface texture. The peak density, S_pd_, is calculated by dividing the number of peaks by the unit area, and the peak curvature, S_pc_, is the arithmetic mean curvature of significant peaks. A peak is selected as the highest pixel within a 16 by 16 nearest neighbors. These two feature parameters can be applied in surface contact models [37].

## 4. Evolution of Micro- and Macro-Texture

**Figure 6:**
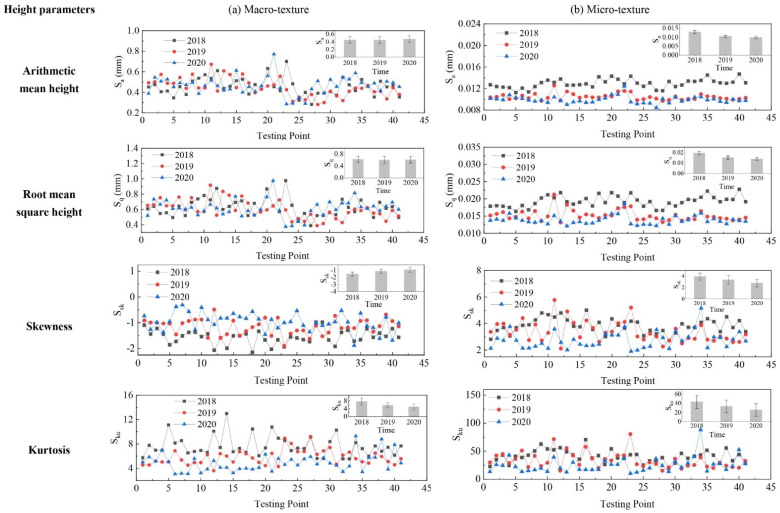
Pavement texture variations via height parameters: (**a**) Macro-texture, and (**b**) Micro-texture.

**Figure 7:**
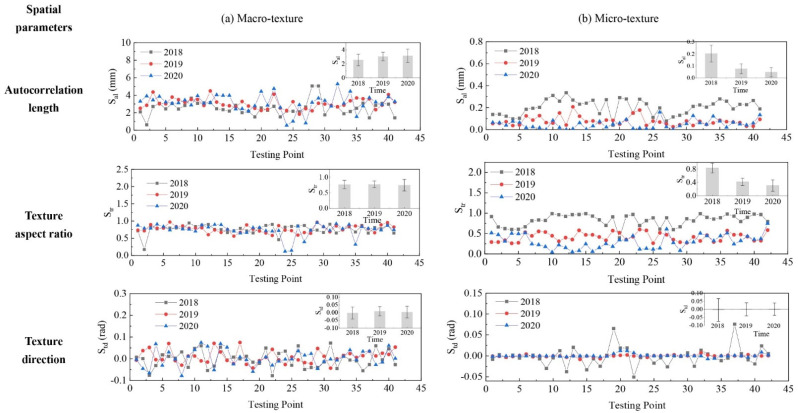
Pavement texture variations via spatial parameters: (**a**) Macro-texture, and (**b**) Micro-texture.

The evolution of pavement micro- and macro-texture was evaluated by comparing 3D areal texture parameters from the three years’ data collection on the field site. The Figure 6, Figure 7, Figure 9, and Figures 11–14 summarize the variations of height, spatial, hybrid, function, and feature parameters for pavement micro- and macro-texture under actual traffic polishing. In each figure, the lines with markers display the actual 3D parameters from each data collection, while the bar chart in the upper-right corner shows the average number and standard deviation of each 3D parameter.

### 4.1. Evolution of Height Parameters

The variations of height parameters for pavement micro- and macro-texture under actual traffic polish are shown in Figure 6. For macro-texture from 2018 to 2020, (1) both S_a_ and S_q_ had no significant distinction in mean value and standard deviation, indicating that traffic polishing was not decreasing the macro-texture’s height. This result corresponds to a previous study that the MPD values tended to remain constant under different polishing cycles [31]; (2) the negative S_sk_ indicates that pavement macro-texture had valley structure; (3) the declined average S_ku_ means that the height variation of surface peaks or valleys was decreasing.

For micro-texture from 2018 to 2020, (1) the S_a_ and S_q_ had an approximate reduction of 20% from 2018 to 2019, and 5% from 2019 to 2020; (2) the S_sk_ were positive and decreased year after year, suggesting the spike structure of micro-texture was decreasing; (3) the S_ku_ was greater than that of macro-texture and gradually reduced, which means the considerable height variation of micro-texture was decreasing as well. The evolution of these height parameters means the spike structure of pavement micro-texture was gradually polished under traffic, as illustrated in Figure 3b.

### 4.2. Evolution of Spatial Parameters

**Figure 8:**
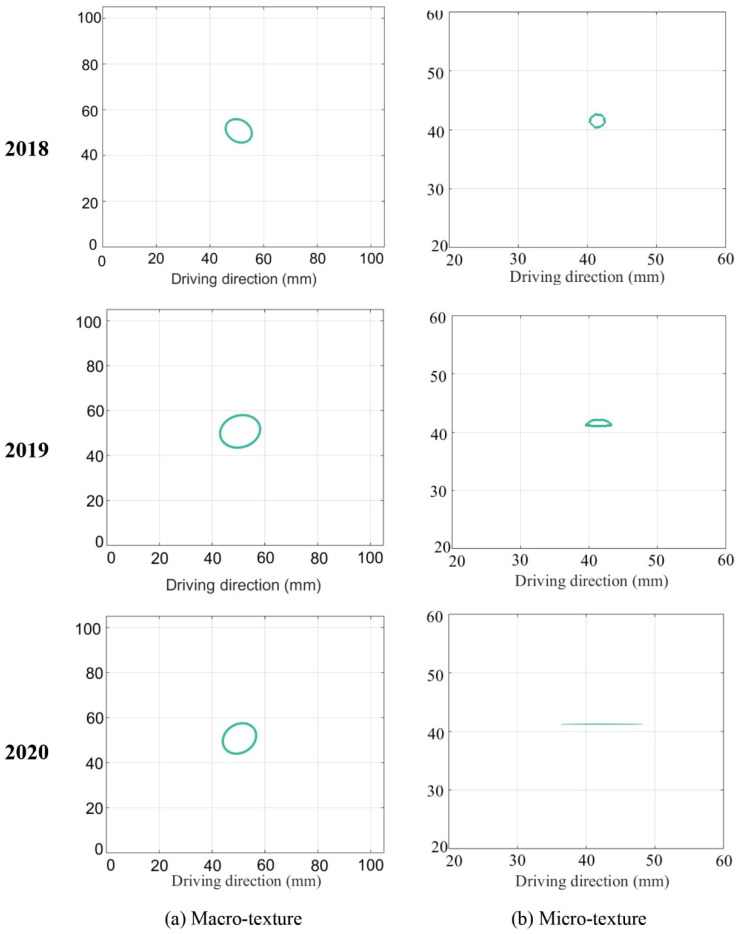
ACF = 0.2 for pavement macro- and micro-texture: (**a**) Macro-texture, and (**b**) Micro-texture.

The variation of spatial parameters for pavement micro- and macro-texture is displayed in Figure 7. For macro-texture from 2018 to 2020, (1) the S_al_ had a 19.5% growth from 2018 to 2019 and stabilized from 2019 to 2020; (2) the S_tr_ was around 0.76 during polish, which means the isotropy of macro-texture was unchanged; (3) the S_td_ was fluctuating around zero. Examples of ACF = 0.2 for macro-texture from 2018 to 2020 are shown in Figure 8a: the shape was stable, meaning the spatial characteristics of macro-texture were not changed from 2018 to 2020 under traffic polish.

For micro-texture from 2018 to 2020, (1) the S_al_ had a descent of 62.3% from 2018 to 2019, and 36.7% from 2019 to 2020; (2) the S_tr_ decreased year after year, indicating the texture changed from isotropic to anisotropic under traffic polishing; (3) the S_td_ was fluctuating around zero, and its deviation decreased year after year. As shown in Figure 8b, the shape of ACF = 0.2 was round in 2018 and became long and thin in 2020, which corresponded to S_tr_ = r_min_/r_max_ decreased from 1.0 to 0.

The spatial evolution of micro-texture can be seen intuitively from Figure 3b. The micro-texture asperities were isotropically distributed in 2018, corresponding to S_tr_ = 1. The micro-texture asperities were anisotropic distributed along the driving direction: stripes appeared in 2020, and the S_tr_ equals 0. Thus, the spatial parameters successfully characterize how pavement micro-texture evolved from isotropic to anisotropic along driving direction under traffic polish.

### 4.3. Evolution of Hybrid Parameters

**Figure 9:**
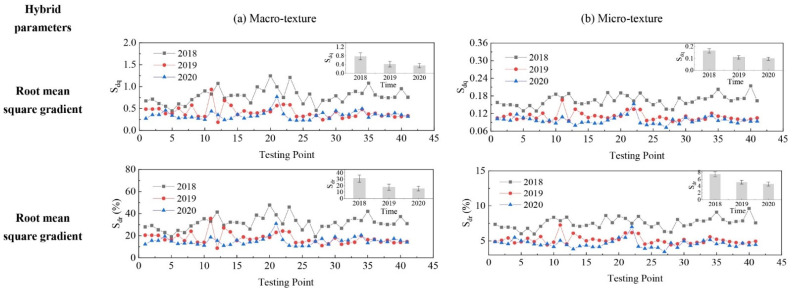
Pavement texture variations via hybrid parameters: (**a**) Macro-texture, and (**b**) Micro-texture.

The variation of hybrid parameters for pavement micro- and macro-texture is displayed in Figure 9. Similar decreasing treads were observed for S_dq_ and S_dr_ from 2018 to 2020. For S_dq_, a reduction of 46.1% and 32.8% were observed for macro- and micro-texture from 2018 to 2019, and another 16.0% and 11.5% of reduction were observed for macro- and micro-texture from 2019 to 2020. As S_dq_ is getting closer to 0, it means the texture surface is getting close to flat under traffic polish with angular slope decreased. For S_dr_, reductions of 43.4% and 32.8% were observed for macro- and micro-texture from 2018 to 2019, and another 16.0% and 113.4% of reduction for macro- and micro-texture from 2019 to 2020. The evolution of hybrid parameters suggests that the steepness and the developed interfacial area of pavement micro- and macro-texture were decreased year after year under traffic polish.

### 4.4. Evolution of Function Parameters

**Figure 10:**
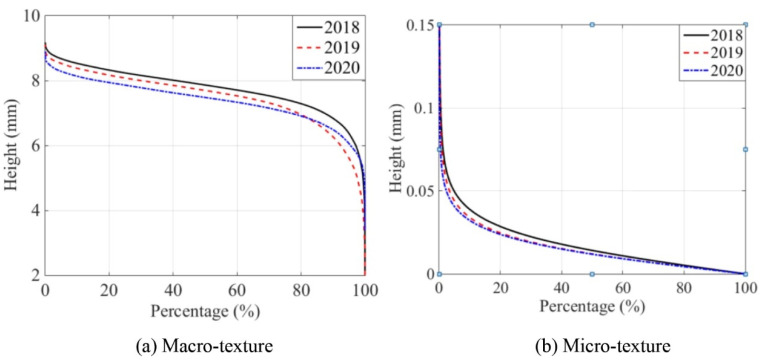
Cumulative height distribution curve of pavement macro- and micro-texture: (**a**) Macro-texture, and (**b**) Micro-texture.

Under traffic polishing, the peak and valley of pavement texture change over time. The cumulative height distribution curve of pavement texture provides an ideal tool to visualize how the texture profile changes due to polishing. Figure 10 shows examples of cumulative height distribution curves for macro- and micro-texture over the years. For example, for macro-texture, the material ratio corresponding to height 8 mm were 41.0% in 2018, 30.1% in 2019, and 16.5% in 2020; for micro-texture, the material ratio corresponding to height 0.05 mm were 4.9% in 2018, 3.4% in 2019, and 2.4% in 2020. This implies that the material of pavement texture was worn due to traffic polish.

Notably, the cumulative height distribution curve of macro-texture in 2019 was lower than that of 2018. It means that the texture material was worn, and the texture valley was increased from 2018 to 2019, which should correspond to the bitumen removal process. The cumulative height distribution curve of macro-texture in 2020 was lower at the peak layer and core layer but higher at the valley layer than that of 2019. This phenomenon illustrates that the upper part of macro-texture was removed by traffic polishing and field environmental erosion, and the valley void collected dust, debris, or chipping under traffic polishing. Besides, micro-texture’s cumulative height distribution curve was getting lower year after year, suggesting micro-texture was consistently polished by traffic.

#### 4.4.1. Evolution of Material Ratio Parameters

**Figure 11:**
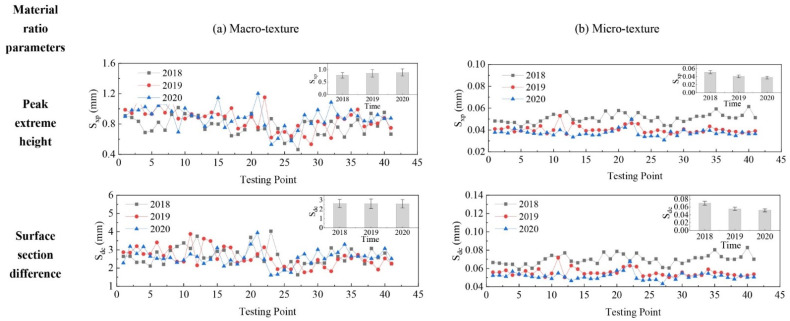
Pavement texture variations via material ratio parameters: (**a**) Macro-texture, and (**b**) Micro-texture.

The variation of material ratio parameters for pavement micro- and macro-texture is displayed in Figure 11. For macro-texture from 2018 to 2020, (1) the S_xp_ slightly increased from 2018 to 2019 and remained stable after the second polish year; (2) the S_dc_ kept almost unchanged. For micro-texture from 2018 to 2020, (1) the S_xp_ had a 20% decrement from 2018 to 2019 and another 7.5% decrement from 2019 to 2020; (2) the S_dc_ decreased by20% and 6.9%, respectively, after the first and second years of polishing. The material ratio parameters of micro-texture changed more than that of macro-texture by traffic polish, suggesting traffic polish mainly affects materials of micro-texture.

#### 4.4.2. Evolution of Stratified Parameters

**Figure 12:**
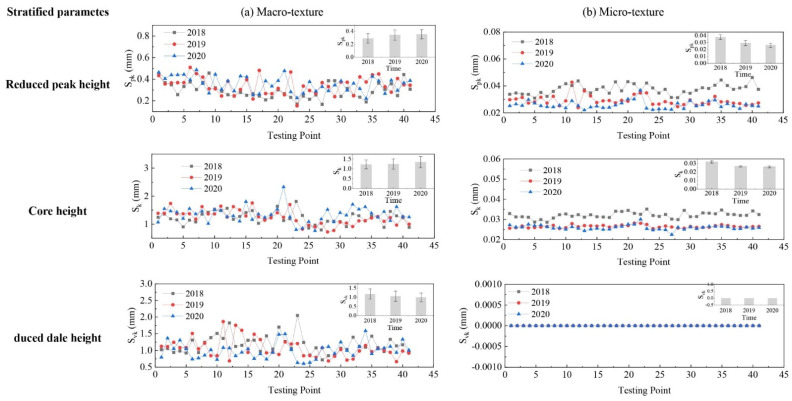
Pavement texture variations via stratified parameters: (**a**) Macro-texture, and (**b**) Micro-texture.

The variation of stratified parameters for pavement micro- and macro-texture is displayed in Figure 12. For macro-texture from 2018 to 2020, (1) the S_pk_ increased by 17.6% from 2018 to 2019 and remained unchanged roughly after the second year of polishing, indicating that the peak layer remained unchanged after the bitumen layer was removed; (2) the S_vk_ of macro-texture decreased by 10% and 5.7% for each polishing year, which implies that the valley structure of macro-texture was gradually filled by dust, debris, or residue under traffic polishing; (3) the S_k_ showed minor variance after two years of traffic polish, which means the core layer of macro-texture was stable under traffic polish. Therefore, the variation of stratified parameters for macro-texture reveals that the traffic polish mainly affects the peak and valley layers but not the core layer of pavement macro-texture.

For micro-texture from 2018 to 2020, (1) the S_pk_ and S_k_ had significant decrement after the first year’s polish and minor change after the second year’s polish; (2) the mean value and standard deviation of micro-texture S_vk_ was almost zero, because the dale stratification did not exist in the cumulative height distribution curve of micro-texture, as shown in Figure 10b. It means traffic polish affects peak, core, and valley layers of pavement micro-texture.

#### 4.4.3. Evolution of Volume Parameters

**Figure 13:**
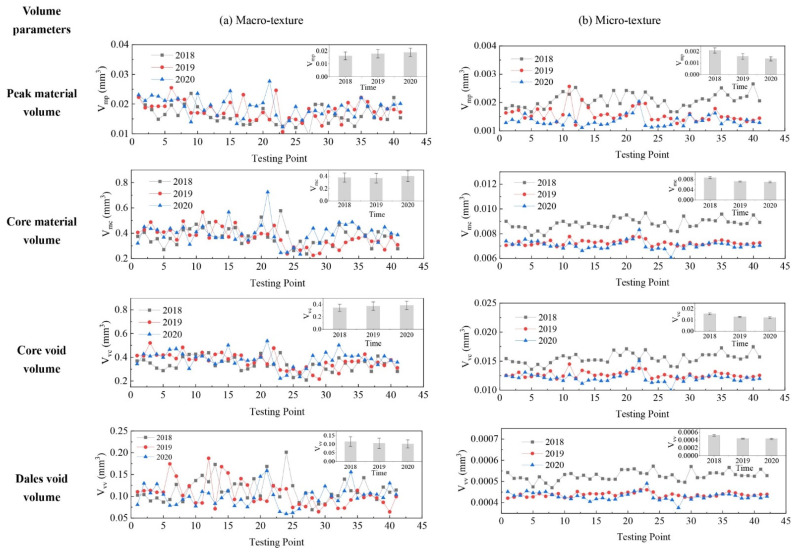
Pavement texture variations via volume parameters: (**a**) Macro-texture, and (**b**) Micro-texture.

Figure 13 shows the variation of volume parameters in three years for pavement micro- and macro-texture. For macro-texture from 2018 to 2020, (1) the V_mp_ increased 9.9% and 5.9% after each polishing year, suggesting more material from the peak layer was exposed under traffic polish; (2) the V_mc_ and V_vc_ remained unchanged, indicating the material and void volume of core layer was unaffected by traffic polish; (3) the V_vv_ slightly decreased 8.5% and 3.9% sequential under traffic, meaning the void volume of valley layer was gradually reduced by collecting dust, debris, or residue under traffic polishing.

For micro-texture from 2018 to 2020, (1) the V_mp_ decreased by 42.9% and 14.0%; (2) the V_mc_ decreased by 16.7% from 2018 to 2019, and changed minor (2.9%) after the second year’s traffic polish; (3) the V_vc_ had a large descend of 17.8% from 2018 to 2019, and minor change (4.7%) from 2019 to 2020; (4) the V_vv_ also had consecutive drops of 16.0% and 1.9%. This result implies that traffic polish affects the material and void volume of pavement micro-texture.

Therefore, the volume parameters suggest that traffic polish influences pavement macro-texture in the following aspects: (1) exposed more material from the peak layer into contact; (2) filled up the valley layer with dust, debris, or residue; (3) had a minor impact on the core layer. Additionally, traffic polish consistently reduced the height or volume of pavement micro-texture peak, core, and valley layers.

### 4.5. Evolution of Feature Parameters

**Figure 14:**
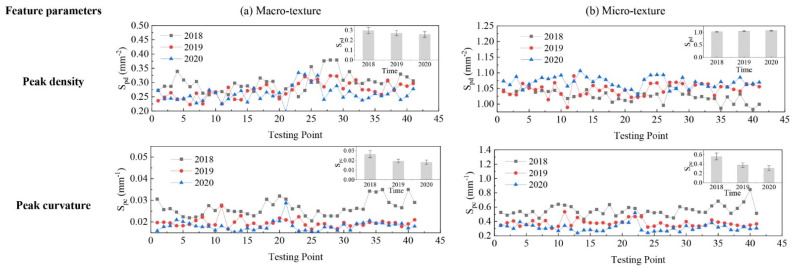
Pavement texture variations via feature parameters: (**a**) Macro-texture, and (**b**) Micro-texture.

The evolution of feature parameters for macro- and micro-texture is shown in Figure 14. Generally, the S_pd_ of macro-texture had a tiny descend of around 5%, which means the number of contact peaks was reduced by abrasion. The average number of S_pc_ dropped 26.3% after the first year’s polish, corresponding to the removal of the bitumen layer and fine aggregate. Then the S_pc_ had only a 7% drop from 2019 to 2020, because the coarse aggregate in pavement structure was gradually exposed and was harder to get worn than bitumen layer under traffic polish.

Unlike the macro-texture, the S_pd_ of micro-texture was slightly increased year after year, as displayed in Figure 14b. The enlarged micro-texture in Figure 3b also shows more peaks existed on micro-texture over time due to traffic polish. The coarse aggregate exposure from 2018 to 2019 and the new micro-texture generated in the wearing process of coarse aggregates from 2019 to 2020 may contribute to the increased S_pd_. However, the S_pc_ was lessening by 33% and 17% after each year’s polish, which means the pavement micro-texture was gradually rounded by polish.

## 5. Conclusions

This paper applies 3D areal parameters to investigate asphalt pavement micro- and macro-texture evolution under actual traffic polish and environmental conditions. The portable 3D laser scanner LS-40 collected high-resolution 3D pavement texture data from predefined locations on a field site in 2018, 2019, and 2020, respectively. The obtained LS-40 data was decomposed into pavement micro- and macro-texture data sets to calculate 3D areal texture parameters. A total number of twenty parameters under five categories (height, spatial, hybrid, functional, and feature) were calculated to study the evolution of pavement micro- and macro-texture under actual traffic polish. The conclusions are summarized as follows:

1. the traffic polish and environmental conditions change the pavement micro-texture as follows: (1) the spike structure was gradually shrunk; (2) the spatial characterization evolved from isotropic to anisotropic; (3) the steepness and the developed interfacial area were decreased; (4) the height or volume of the peak, core, and valley layers reduced consistently; and (5) the peak density increased but peak curvature decrease.
2. the traffic polish and environmental conditions change the pavement macro-texture as follows: (1) the had valley structure and the height variation of surface peaks decreased; (2) the spatial characteristics were not changed under traffic polish; (3) its steepness and the developed interfacial area were decreased; (4) the material of the peak layer removed, and the valley layer filled up with dust, debris, or residue, and (5) the peak density and peak curvature were all decreased.

The results demonstrate the advantage of 3D areal parameters to describe the evolution characterization of pavement micro- and macro-texture under traffic polish. However, this paper only recorded texture data from one asphalt mixture in three years. Thus, it is expected that more asphalt pavement texture categories could be collected for a longer time frame in a future study to understand how traffic polish affects pavement micro- and macro-texture for different pavements. Furthermore, the relationship of texture wear and skid resistance should be studied in the future as well.

## Figures and Tables

**Table 1:** Summary of 3D areal parameters.

Category		Parameters	Unit
**Height Parameters**		arithmetic mean height (S_a_)	mm
		root mean square height (S_q_)	mm
		skewness (S_sk_)	-
		kurtosis (S_ku_)	-
**Spatial** **Parameters**		autocorrelation length (S_al_)	mm
		texture aspect ratio (S_tr_)	-
		texture direction (S_td_)	rad
Hybrid Parameters		root mean square gradient (S_dq_)	-
		developed interfacial area ratio (S_dr_)	%
**Function** **Related** **Parameters**	Material Ratio Parameters	peak extreme height (S_xp_)	mm
		surface section difference (S_dc_)	mm
	Stratified Parameters	reduced peak height (S_pk_)	mm
		core height (S_k_)	mm
		reduced dale height (S_vk_)	mm
	Volume Parameters	peak material volume (V_mp_)	mm^3^
		core material volume (V_mc_)	mm^3^
		core void volume (V_vc_)	mm^3^
		dales void volume (V_vv_)	mm^3^
**Feature Parameters**		peak density (S_pd_)	mm^−2^
		peak curvature (S_pc_)	mm^−1^

## Data Availability

Not applicable.
